# Control of initial steps of ciliogenesis by protein kinases

**DOI:** 10.1186/2046-2530-4-S1-O4

**Published:** 2015-07-13

**Authors:** R Carvalho, W Wang, G Pereira

**Affiliations:** 1 German Cancer Research Centre (DKFZ), DKFZ-ZMBH Alliance and University of Heidelberg, Heidelberg, Germany

## 

Cilia are microtubule-based organelles present on the surface of most vertebrate cells. The formation of the primary cilia requires the mother centriole, which is the older of the two centrioles, to convert into the ciliary basal body. Although recent research has begun to shed light onto the molecular composition of the cilium, the regulation of ciliogenesis is just beginning to be understood. To get insight on the molecular mechanisms that regulate basal body formation, we performed a high-throughput RNA interference screen to identify protein kinases required for ciliogenesis. Of the novel kinases we identified, we are characterizing the function of the microtubule associated/affinity regulating kinase 4 (MARK4), and tau-tubulin kinase 2 (TTBK2). We show that MARK4 and TTBK2 are both required to initiate axoneme extension and to promote the removal of the inhibitory protein complex composed of CP110/Cep97. Together, our data indicate that cilia formation is a highly regulated process, which requires the concerted action of protein kinases that regulate the transition from the mother centriole into the basal body.

